# Temporal Processing of Vibratory Communication Signals at the Level of Ascending Interneurons in *Nezara viridula* (Hemiptera: Pentatomidae)

**DOI:** 10.1371/journal.pone.0026843

**Published:** 2011-10-28

**Authors:** Maja Zorović

**Affiliations:** Department of Entomology, National Institute of Biology, Ljubljana, Slovenia; Cajal Institute, Consejo Superior de Investigaciones Científicas, Spain

## Abstract

During mating, males and females of *N. viridula* (Heteroptera: Pentatomidae) produce sex- and species-specific calling and courtship substrate-borne vibratory signals, grouped into songs. Recognition and localization of these signals are fundamental for successful mating. The recognition is mainly based on the temporal pattern, i.e. the amplitude modulation, while the frequency spectrum of the signals usually only plays a minor role. We examined the temporal selectivity for vibratory signals in four types of ascending vibratory interneurons in *N. viridula*. Using intracellular recording and labelling technique, we analyzed the neurons' responses to 30 pulse duration/interval duration (PD/ID) combinations. Two response arrays were created for each neuron type, showing the intensity of the responses either as time-averaged spike counts or as peak instantaneous spike rates. The mean spike rate response arrays showed preference of the neurons for short PDs (below 600 ms) and no selectivity towards interval duration; while the peak spike rate response arrays exhibited either short PD/long ID selectivity or no selectivity at all. The long PD/short ID combinations elicited the weakest responses in all neurons tested. No response arrays showed the receiver preference for either constant period or duty cycle. The vibratory song pattern selectivity matched the PD of *N. viridula* male vibratory signals, thus pointing to temporal filtering for the conspecific vibratory signals already at level of the ascending interneurons. In some neurons the responses elicited by the vibratory stimuli were followed by distinct, regular oscillations of the membrane potential. The distance between the oscillation peaks matched the temporal structure of the male calling song, indicating a possible resonance based mechanism for signal recognition.

## Introduction

Substrate-borne vibratory communication plays an important role during long-range calling and short-range courtship in many small plant dwelling insects [1], [2], [3]. In order to find a conspecific mate, insects have to recognize and localize the emitter of the communication signals. The frequency spectra of the signals are tuned to the resonant frequencies of the host plants, ensuring maximum efficacy of signal transmission [4]. Consequently, the dominant frequency of signals produced by the vibratory mechanism usually lies below 200 Hz and above 50 Hz. Recognition of a conspecific signal is therefore mainly based on the temporal pattern, while the spectrum of the signals normally plays a minor role. This is reflected in the enormous diversity of species-specific and stereotyped song patterns found in most vibrationally communicating insects, whereas the spectra, especially of related species, usually differ much less from each other, as the vibrations producing systems are usually very similar [5].

Song pattern recognition including temporal selectivity for insect substrate-borne vibratory signals has been investigated mainly at the behavioural level in Hemiptera, suborders Auchenorrhyncha [6], [7], and Heteroptera [8], [9], [10]. However, this ancient and primitive communication system has long been overlooked and understudied [11], and the neural mechanisms underlying the song pattern recognition processes are still best analyzed in insects that communicate primarily using air-borne sound. These include mainly crickets and acridid grasshoppers with some information available also on katydids, cicada, moths and other groups [12]. Although some peripheral specializations allow the frequency filtering of the species-specific signals, the final decisive steps of temporal recognition are apparently accomplished in the CNS and in particular in the brain [13], [14], [15], [16], [17]. Nonetheless, some studies suggest a certain level of temporal filtering also at the level of the ascending interneurons [18], [19], [20], [21].

While in crickets there are apparently only two auditory ascending interneurons, AN1 and AN2 [22], [23], [24], in the auditory system of acridid grasshoppers there is a group of at least 25 such interneurons that carry information from the thoracic ganglia towards the brain [25]. While most insect auditory pathways are characterized by two major inputs (one pair of tympanic ears), the vibratory system is a far more complex, multiple-input system. A set of various vibratory receptors is present in each of the legs, including joint chordotonal organs, the subgenual organ and campaniform sensillae. Additionally, when in contact with the substrate, the vibrations can also be perceived by the Johnston's organ in the antennae. In such systems, at least 10 vibratory interneurons have been described so far that ascend from the central ganglion towards the brain [26], [27], [28], [29].

The other important difference between the two systems lies in the physical characteristics of the communication signals themselves and their dependence on the transmission medium. In case of the air-borne sound spreading spherically from a point source, the intensity decreases with the distance from the source more or less uniformly. However, in solids, molecules can support vibrations in different directions; hence a number of different types of sound waves are possible. Waves can be characterized in space by oscillatory patterns that are capable of maintaining their shape and propagating in a stable manner. The propagation of waves is often described in terms of what are called “wave modes”. Some invertebrates may for example exploit compressional (scorpions; [30]) or transverse boundary waves on sand surfaces (fiddler crabs; [31]). It was shown that the nature of the waves elicited by the insects that communicate using substrate-borne vibrations on plants is that of the bending waves [32]. Their main characteristics involve dispersive propagation, meaning that higher frequencies travel faster than low frequencies. Therefore, during propagation, signals are transformed into frequency modulated sweeps of prolonged duration. Additionally, reflections of the signal occur at the root and the top of the plant leading to standing wave conditions in stems and other rod-like plant structures, with nodes (points with low intensity of the signal) and internodes (points with high signal intensity). Due to dispersive propagation, the nodes and the internodes occur at different points along the plant for different frequency peaks. The system is further complicated by the occurrence of various branching points, on which the searching insect has to orientate towards the source of vibrations. Based on all these differences between the two systems, we hypothesized that the evolutionary primitive system for processing substrate-borne vibratory signals differs in organization, complexity and/or mechanisms from the one developed later in insects communicating mainly with air-borne sound signals.

In this study we aimed to investigate whether any song temporal processing/selectivity occurs at the level of the ascending neurons in a model insect for substrate-borne vibratory communication. Since the mating behaviour of the green stink bug *Nezara viridula* [1], [33] and transmission properties of their host plants [3], [4] have been extensively studied, we could correlate our findings with the temporal parameters of their natural vibratory communication signals. Additionally, the functional and morphological properties of their vibratory receptor neurons [34], [35] and several ascending, local and descending neurons in the thoracic ganglia have been described [26], [27]. *N. viridula* is therefore a suitable model species to study the neural principles and mechanisms of the species-specific mate recognition based on vibratory signalling.

Vibratory communication in *N. viridula* starts with the emission of the female calling song (FCS) during which the female remains stationary on the plant. The male, on the other hand, emits its signals while actively searching for the female [36]. Usually, the FCS induces a chain of events including increased male pheromone emission [37], male activation, its searching behaviour and approach to the female, accompanied by the male calling (MCS) and/or courtship songs (MCrS). The continuous emission of the FCS enables the males to locate the females [36], [38], while the occasional emitting of their vibratory signals stimulates continuous emission of the FCS at a stable repetition rate [39]. The mean recorded durations for the FCS of the Slovenian population of *N. viridula* were 720±37 ms for the PD and 1912±93 ms for the ID [10]. The study [10] used behavioural tests to investigate the preference of the males for the temporal parameters of the FCS. The findings showed that the males responded best to the FCS of the natural duration, while the response significantly decreased below 600 ms (with none of the males responding below 200 ms) and above 1000 ms PD. In case of the ID, the males also showed greatest preference for ID values of the natural song, but tolerated durations of 1500 and up to 3000 ms, with another peak at 7000 ms. It was evident that the males showed stronger preference for the PDs than the IDs. Another feature that was found in the aforementioned study to significantly influence the success of the male searching is the short pre-pulse before the main pulse of the FCS, lasting ca. 130 ms. A similar behavioural study, evaluating the ideal temporal parameters (in the sense of the eventual successful mating) of the male searching song on the continuous emission of the FCS has not been yet been conducted and should be considered in future investigations.

While, to our knowledge, the studies of the vibratory song pattern recognition at the level of central processing are still completely lacking, similar investigations in the field of insect air-borne sound communication were focused mostly on the analysis of the time-averaged spike rates. Coding by the instantaneous spike rate has been almost completely neglected in studies that analyze the activity patterns of central auditory interneurons, although it is fundamental to temporal summation of postsynaptic potentials [20]. It was shown that the peak instantaneous spike rates of the local ON1 cricket neuron are sufficient to explain temporal filtering early on in their auditory pathway [20]. Also, in both the behavioural and the neurophysiological studies of song pattern selectivity in air-borne sound communication, usually only a limited number of PD/ID combinations were used, keeping the duty cycle at 50% and changing only the pulse period (for review see: [16]). In such experiments, the test patterns fall on the diagonal line of the response arrays and do not allow distinguishing between a range of possible temporal filters; therefore we designed the stimulus test sequence with the PD/ID response arrays determined in more detail.

We found evidence for temporal filtering based on signal duration in all neurons tested and little or no influence of the interval duration on the neurons' responses. We discuss the differences between the time-averaged spike count versus the instantaneous spike frequency as a measure for analysing selectivity/filtering properties of the vibratory interneurons. In some neurons of type CG-AC7, each stimulus was followed by the oscillations of the membrane potential; this points to possible resonance mechanism for temporal selectivity at the level of the ascending vibratory interneurons.

## Results

### Morphology

**Figure 1:**
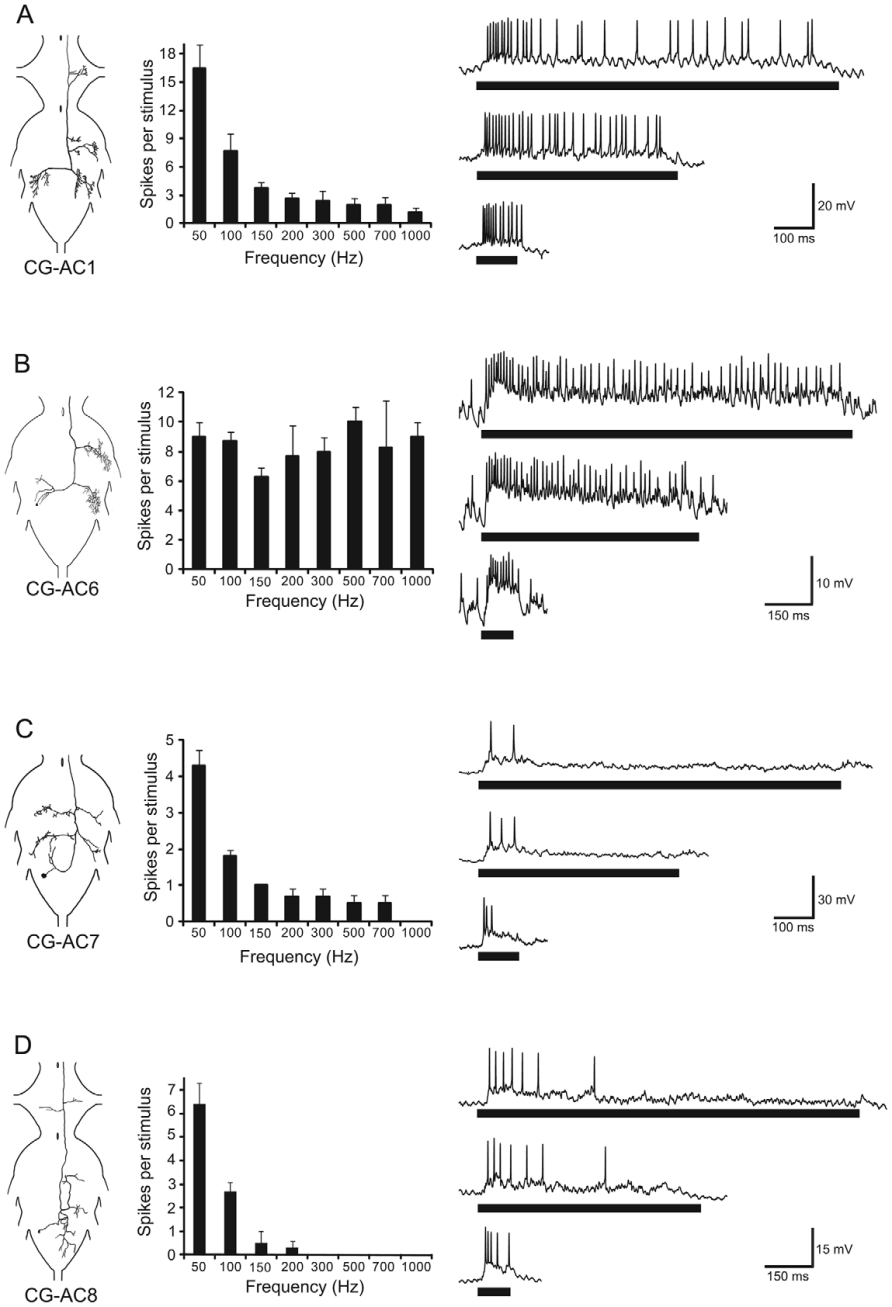
Arborization patterns, frequency tuning and responses of vibratory interneurons CG-AC1, CG-AC6, CG-AC7 and CG-AC8. *Left:* Wholemount drawings of CG-AC1 and CG-AC8 in both the CG and PTG and the CG-AC6 and CG-AC7 in the CG. *Middle:* The histograms in the middle column show the frequency preference of neurons at 20 cm/s^2^ as the number of spikes per 20 ms stimuli of varying frequencies (N = 2–4, n = 3–24; means and SE are shown). The traces of intracellular recordings in the right column show typical responses of neurons to 105 Hz sine wave stimuli of different durations and stimulus intervals. The intensity of all stimuli was 20 cm/s^2^. *Right:* (A) SDs were 100, 500 and 900 ms. ID was 1000 ms in all cases. (B) SDs were 100, 500 and 900 ms. ID was 1500 ms in all cases. (C) SDs were 100, 700 and 1200 ms. ID was 1500 ms in all cases. (D) SDs were 100, 700 and 1200 ms. ID was 1500 ms. Bars below the intracellular recording traces represent the stimuli.

We describe four morphologically distinct types of interneurons (Figure 1A-D, *left*) with the cell body in the metathoracic region of the central ganglion (CG) and axons ascending contralaterally into the prothoracic ganglion (PTG). In CG-AC1 and CG-AC8 the axons pass through the PTG and ascend further anteriorly towards the suboesophageal ganglion (SOG). The morphology and physiological properties of CG-AC1 were described in detail in [27], while CG-AC6, CG-AC7 and CG-AC8 have not been described previously. The cell body of CG-AC6 is located ventrolaterally in the metathoracic segment of the CG, with the dendritic region limited to the ipsilateral part of the CG. The axon ascends contralaterally to the cell body, close to the midline, sending extensively branching collaterals into the dorsal neuropils of the contralateral mid- and hind legs. The cell body of CG-AC7 is located laterally in the anteroventral part of the abdominal segment of the CG. The primary neurite runs anteromedially and branches ipsilaterally into the dendritic region in the ipsilateral neuropile of the hind leg. The axon crosses the midline and turns anteriorly. The axon collaterals branch ipsi- and contralaterally in the dorsal neuropils of the mid- and hind legs. The soma of CG-AC8 is located in the same area as the soma of CG-AC7. The primary neurite runs anteromedially and turns sharply to the dorsal side of the CG immediately after crossing the midline. The neuron branches in the abdominal as well as meta- and mesothoracic segments of the contralateral side of the CG. In the PTG, the neuron sends collaterals ipsi- and contralaterally into the dorsal neuropils of the front legs.

### Physiology

**Figure 2:**
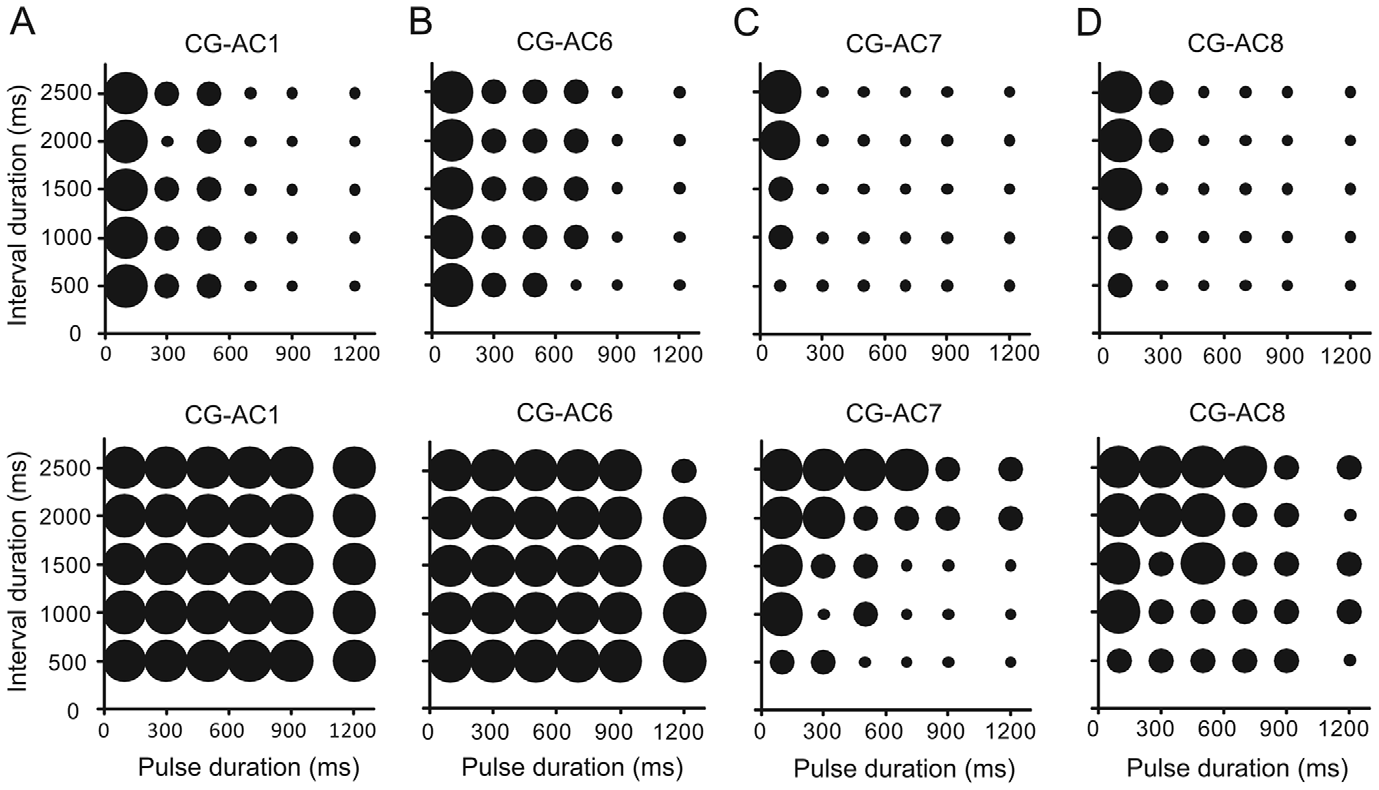
Response arrays from four types of vibratory interneurons. (A) to (D) Mean spike frequency response arrays (*uper row*) and peak instantaneous spike frequency response arrays (*lower row*) for CG-AC1 (N = 4, n = 3–6 for every PD/ID combination), CG-AC6 (N = 2, n = 3–6 for every PD/ID combination), CG-AC7 (N = 3; n = 3 for every PD/ID combination) and CG-AC8 (N = 2; n = 3 for every PD/ID combination). Large circles indicate a strong response, medium-sized circles indicate an intermediate response and small circles indicate a weak or no response.

The frequency preference was established at the same stimulus intensity for all neuron types and is shown in the form of histograms as the number of spikes per stimulus (Figure 1A–D, *middle*). We used the stimulus intensity that was shown to be well above threshold for all vibratory interneurons described previously in the thoracic ganglia of *N. viridula*, 20 cm/s^2^ [27]. The dynamics of the response to 105 sine wave stimuli (tonic, phasic, phasic-tonic) is described for each neuron type and the response traces for different PDs at a constant ID are shown (Figure 1A–D, *right*). The temporal selectivity is presented in the form of response arrays, which show the responses of neurons to different PD/ID combinations (Figure 2). The intensity of the responses is shown as either time-averaged spike rate (Figure 2, *upper row*) or the peak instantaneous spike rate (Figure 2, *lower row*).

### CG-AC1

The hyperbolic decrease in the number of spikes per stimulus for frequencies ranging from 50 to 1000 Hz indicates a high preference of CG-AC1 for the lowest frequency tested (Figure 1A, *middle*). An increase in the stimulus frequency from 50 to 100 Hz resulted in a large drop in the number of spikes; from 16.4±2.6 to 7.7±1.8 (N = 4, n = 18), respectively. The intracellular recordings (Figure 1A, *right*) show an excitatory phasic-tonic response. The increase of PD from 100 ms (*lower trace*) to 500 ms (*middle trace*) and 900 ms (*upper trace*) at constant ID of 1000 ms shows increased adaptation of the response with a marked decrease in the spike rate, especially in the tonic part of the response after the first 120–150 ms from the pulse onset. Response array displaying the mean spike rates (Figure 2A, *upper array*) reveals low pass filtering for PDs and no selectivity for IDs. The peak instantaneous spike rates of CG-AC1 were not affected by changes in either PD or ID values (Figure 2A, *lower array*).

### CG-AC6

Neurons of this group showed no frequency preference in the range between 50 and 1000 Hz (Figure 1B, *middle*). The traces of intracellular activity (Figure 1B, *right*) show excitatory responses to vibratory stimuli, with action potentials riding on top of the EPSPs. The responses are phasic-tonic, albeit with a less pronounced phasic part than type CG-AC1. We observed no marked adaptation in the tonic part of the response with the increase in PD from 100 ms (*lower trace*) via 500 ms (*middle trace*) to 900 ms (*upper trace*). The response array (Figure 2B, *upper array*) shows a similar, albeit slightly broader low pass filtering for PDs as in CG-AC1 with the strongest responses at 100 ms, but only when the stimulus intensity is given as the mean spike rate. The changes in ID did not affect either the mean or the peak instantaneous spike rates (2B, *lower array*).

### CG-AC7

**Figure 3:**
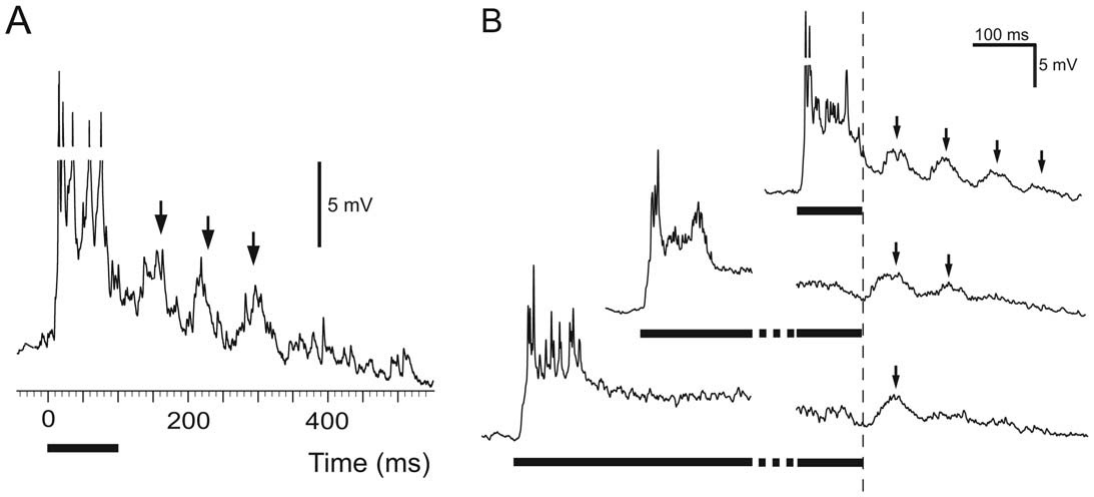
Oscillations in the membrane potential evoked by sine-wave stimuli of 105 Hz carrier frequency in CG-AC7. (A) An intracellular recording of a single response to a 100 ms stimulus. Oscillations of the membrane potential are indicated by arrows. Note that the spikes are not shown in whole; their maximum amplitude was 30 mV. (B) Waveform averages of 17 responses to the different PDs: 100 ms (*upper trace*), 700 ms (*middle trace*) and 1200 ms (*lower trace*). The periodical graded potentials (*arrows*) following the stimulus occurred in ca. 80 ms intervals. The IDs did not affect the occurrence or the size of the oscillations. Note that in the upper trace the spikes are not shown in whole, their max. amplitude reached 30 mV.

The isointensity histogram of CG-AC7 (Figure 1C, *middle*) shows strong preference for 50 Hz. Although the neurons responded to stimulus frequencies up to 700 Hz, the response dropped sharply with the increase of frequency from 50 to 100 Hz. The traces of the intracellular recordings (Figure 1C, *right*) show that CG-AC7 neurons responded in a highly phasic manner even to the shortest pulses tested (lower trace, PD = 100 ms). The upper response array in Figure 2C shows strong selectivity towards short PDs combined with weak selectivity towards longer IDs. Similarly, the lower array in Figure 2C shows that the increase in the peak instantaneous spike rates was correlated with a decrease in PD and an increase in ID. In some CG-AC7 neurons, we observed post-stimulus membrane potential oscillations at regular 80 ms intervals, with the first peak positioned at ca. 50 ms after the end of the pulse (Figure 3A, *arrows*). At PD 100 and 300 ms, each pulse was followed by 3 to 4 such graded membrane potentials of up to 7 mV in size (Figure 3B, *upper trace*). At higher PDs their number decreased (Figure 3B, *middle trace*) and at PD 1500 ms only a single, although more prominent peak was observed (Figure 3B, *lowest trace*).

### CG-AC8

CG-AC8 neurons showed a high preference for the lowest frequency tested, 50 Hz (Figure 1D, *middle*). The number of spikes decreased sharply with the increase of the stimulus frequency to 100 Hz, while there was barely any response left at 150 and 200 Hz (1–2 spikes per stimulus); no spikes were elicited above 200 Hz. The intracellular recordings shown in Figure 1D (*right*) show an excitatory phasic-tonic response to a 100 ms stimulus (*lower trace*) with a distinct phasic component. The responses to the 900 ms and 1200 ms stimuli (*middle and upper traces*) are phasic in the sense that the spiking is limited to the beginning of the stimulus, however, the spike frequency does not change much during the stimulus and the neurons mainly stop spiking at ca. 200 ms after the onset of the stimulus. The upper response array in Figure 2D shows a somewhat weaker selectivity towards the shortest PDs and longest IDs than CG-AC7. Similarly, the increase in the peak instantaneous spike rates (Figure 2D, *lower array*) was correlated with a decrease in PD and an increase in ID, like in CG-AC7, albeit the changes were not as substantial.

## Discussion

We described song pattern selectivity of four types of ascending vibratory interneurons in the central ganglion of the southern green stink bug *N. viridula* (Heteroptera: Pentatomidae) by evaluating their responses to a range of different PD/ID combinations using time-averaged and peak instantaneous spike rates. In some CG-AC7 neurons, the stimuli were followed by the membrane potential oscillations at regular intervals, which were close to the duration of the natural male calling song (MCS). This finding points to potential resonance based mechanism for signal recognition.

The cell bodies of all neuron types described are located in the or near to the region of the hind leg neuropile in the central ganglion (CG). For neurons CG-AC1 and CG-AC8 the stainings revealed the axons passing through the prothoracic ganglion towards the suboesophageal ganglion, while in CG-AC6 and CG-AC7 we only have evidence that the axons leave the CG contralaterally. Therefore it was not established if any of the neurons described here actually carry the information on vibratory communication signals directly to the brain. Based on their morphology and physiological properties, they are all, however, a part of the ascending vibratory pathway.

The isointensity frequency tuning evaluated by the number of spikes per stimulus revealed that except CG-AC6, all neurons (as most of the ascending neurons described in the previous study [27]) have a maximum response at the lowest frequency tested, 50 Hz, and that with the increase in signal frequency the response dropped rapidly. Only CG-AC6 showed no frequency preference in the range from 50 to 1000 Hz. The dominant peak of most *N. viridula* and other investigated stink bug male and female communication signals lies between 85 and 120 Hz [3], [42]; this would explain why the neurons are mostly tuned to frequencies below 150 Hz.

While two neuron types, CG-AC1 and CG-AC6 responded phaso-tonically to all pulse durations tested, the CG-AC7 and CG-AC8 responded either phasically to all pulse durations or phaso-tonically only at the shortest pulses and phasically at pulse durations above 100 ms. These differences in their response modes were reflected in their song pattern selectivity (Figure 2), the differences are particularly evident in the peak instantaneous spike rate arrays where CG-AC1 and CG-AC6 show no selectivity for either PD or ID values.

Although some studies carried out on the ascending auditory interneurons suggest that the neurons are not sex-specific, at least not morphologically, there is some evidence that the same neuron may serve different functions in males and females [18], [43]. Because we only used female *N. viridula* in our experiments, we will first assume that the temporal selectivity of a given neuron type is a sex-specific trait, although similar experiments have yet to be carried out using males to fully support (or, indeed, disprove) this assumption.

The values for the PD and ID of the male calling vibratory songs were measured to be between 72±14 and 145±43 ms for the PD and 59±43 and 324±122 ms for the ID for different populations of *N. viridula* [42], [44]. The PD of the male courtship song was, however, much longer, ca. 3000 ms in duration, while there is no data available on its ID.

The upper row of arrays in Figure 2 that show the response intensity as time-averaged number of spikes, exhibit no major differences between different cell types. All neurons seem to be low pass filters for the PDs while there is little or no influence of the IDs on the response intensity. The narrowest selectivity is shown for CG-AC7 and CG-AC8 and at the same time these are the only neuron types where there is also some obvious ID selectivity present with the responses rising with the increase in IDs. The PD low-pass selectivity is most narrow in CG-AC6, with the strong and middle responses up to PDs of 700 ms. These arrays fit well with the duration of the natural male calling song, but not with the male calling song IDs, which are usually below 500 ms. Weak or no influence of the IDs may also be due to the fact that the male songs are not emitted as continuously and rhythmically as the FCS and therefore their pulse repetition time might possibly be less relevant behaviourally.

However, if we look at the peak instantaneous spike rates (the lower response arrays), which have in the past been neglected in similar studies, the situation changes dramatically. In this case, CG-AC1 and CG-AC6 show no selectivity at all, for either the PD of the ID values. CG-AC7 and CG-AC8, however, display similar song pattern selectivity, more pronounced in CG-AC7. Both neuron types favour the PD/ID combinations above the arrays' constant duty cycle diagonal; the response increases with deceasing PDs and increasing IDs, being strongest at PDs of 100 and 300 ms and IDs of 2000 and 2500 ms. While the PD selectivity fits well with the measured values for the male calling song [42], [44], they again do not match the natural IDs. The selectivity for the values of the male courtship song (see above) cannot be explained by none of the neurons presented in this paper.

Let us assume, however, that the temporal selectivity of these vibratory neurons is not necessarily sex-specific; based on the behavioural study from 2011 [10], we might then expect to find band pass selectivity for PDs with maximum responses at 700 ms and high pass filtering for IDs above 1500 ms. The mean recorded durations for the FCS of the Slovenian population of *N. viridula* in the aforementioned study were 720±37 ms and 1912±93 ms for the PD and ID values, respectfully. Behavioural tests were used to investigate the preference of males for the temporal parameters of the FCS. The findings showed that the males responded best to the FCS of the natural duration, while the response significantly decreased below 600 ms (with none of the males responding below 200 ms) and above 1000 ms PD. In case of the ID, the males also showed greatest preference for ID values of the natural song, but tolerated durations of 1500 and up to 3000 ms, with another peak at 7000 ms. It was evident that the males showed stronger preference for the PDs than the IDs.

Based on our results, we may speculate that either the neurons are sex-specific, with a greater selectivity for PDs than IDs, and that some temporal filtering occurs before the information reaches the brain, or, in the case of CG-AC1 and CG-AC6 (peak spike rate arrays), that some ascending neurons simply transfer the song pattern from the periphery to higher processing centres without any song processing taking place in the early phases. The peak spike arrays of CG-AC7 and CG-AC8 and the time-averages spike number arrays of all the neuron types, however, show some temporal filtering, although it only matches the natural male vibratory signals in their PDs, but not the IDs.

Various models have been proposed and investigated in sound-communicating insects, among them high, low, or band-pass filters, autocorrelation and quite recently, a new concept was added to this list; the mechanism based on neural resonance that was shown in the behavioural experiments on the katydid *Tettigonia cantans* [45]. The neural oscillations found in type CG-AC7 point towards a similar resonance based mechanism. Previously, a similar phenomenon was observed also in a brain auditory interneuron in the cricket *Telleogryllus oceanicus* [16].

Overall, we have shown that certain temporal selectivity takes place at the early stages of neural processing of the vibratory substrate-borne communication signals and that the parameters that we choose to analyse can greatly influence our interpretation of the results, as was shown previously [20]. More elaborate behavioural and neurophysiological investigations will be needed, however, to find the mechanisms underlying the temporal pattern recognition in insects communicating via substrate-borne vibratory signals.

## Materials and Methods

### Animals and preparation

*Nezara viridula* (L.) (Hemiptera: Pentatomidae) nymphs and adults were collected in the Slovenian coastal region. The colony was kept in the laboratory at 24–28°C, relative humidity of 60–70% and 16·h:8·h L:D cycle. The experiments were performed on adult females at least one week after their final moult.

For dissection the bugs were fixed dorsal side up on a U-shaped holder using mixture of beeswax and resin. The wings, pronotum and scutellum were removed. The exposed flight muscles and the underlying adipose tissue were excised to reveal the thoracic ganglia. The tissue was immediately covered with insect saline (ionic composition in mmol l^−1^: 140·NaCl; 10·KCl; 4 CaCl_2_, 4 NaHCO_3_, 6 NaH_2_PO_4_). The vibratory stimuli were delivered to the tarsi of all six legs using a minishaker (Bruel & Kjær; Nærum, Danemark; type 4810) with a steel stud screwed on top of it (Bruel & Kjær, YQ2960). A 5×5 mm steel platform was glued on top of the stud. The U-shaped holder with the animal was lowered until the legs were resting on the platform in a natural position [27]. A spoon-shaped steel platform was used to stabilize the central ganglion (fused mesothoracic, metathoracic and abdominal ganglia; from here on referred to as CG), while it also served as a reference electrode.

### Electrophysiology

Experiments took place on an anti-vibration steel table in a Faraday cage, at rearing temperature and humidity. Intracellular recordings were made from the CG using thick-walled borosilicate glass microelectrodes filled with either Lucifer Yellow CH (5% in 0.5 M LiCl, Sigma-Aldrich, St. Louis, MO) or with Neurobiotin (5% in 1 M KAc or KCl, Vector Laboratories Inc., Burlingame, CA). The electrode resistance ranged between 40 and 90 MΏ. The intracellular signal was amplified with Cyto 721 (WPI, Sarasota, FL), digitized with data acquisition interface (Power1401 mk2, Cambridge Electronic Design, Cambridge, UK) and stored on a PC using Spike2 software (Cambridge Electronic Design, Cambridge, UK).

After data collection, the hyperpolarizing current of 3–5 nA was applied to fill the neuron with Lucifer yellow, or, in case of Neurobiotin, the dye was injected using pulses of depolarizing current. The nervous system was dissected out and fixed in 4% formaldehyde in phosphate buffer, pH 7.4; for 1 h at room temperature or overnight in the refrigerator. The ganglia were treated in different ways depending on the dye. Ganglia treated with Lucifer yellow were dehydrated in an ascending alcohol series and cleared in methyl salicilate for photographing. Specimens stained with Neurobiotin were treated by the avidin-biotin method in whole-mounts [40] and processed using the ABC kit PK 6100 followed by intensification with DAB Peroxidase Substrate Kit SK 4100 (both Vector Laboratories Inc., Burlingame, CA). The ganglia were then dehydrated in the alcohol series and cleared in methyl salicilate. The stained neurons were examined by light or fluorescent microscopy using an AxioCam MR camera (Carl Zeiss, Oberkochen, Germany) attached to a microscope (type DMRB/E, Leica Microsystems, Wetzlar, Germany). Drawings were made with graphic tablet G-pen M712X (Genius, KYE Systems Corp., Taiwan).

### Vibratory stimulation

**Figure 4:**
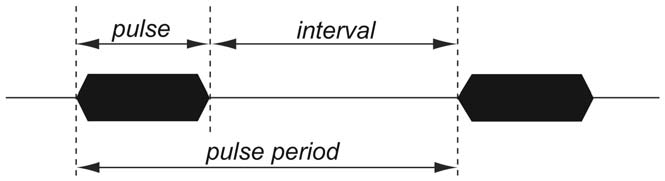
A diagram explaining the terms pulse, interval and pulse period as they are used in the text.

To establish the frequency preference, each neuron was first tested using computer synthesized series of 200 ms long sine wave stimuli with 10 ms rise and fall times, 800 ms interval durations, 50 to 1000 Hz frequency and 20 cm/s^2^ acceleration. Each stimulus frequency was repeated 3–6 times. The second test sequence was designed to determine temporal selectivity of the neurons. It consisted of computer synthesized sine wave pulses of 105 Hz frequency and 20 cm/s^2^ acceleration. Thirty pulse duration/interval duration (PD/ID) combinations (Figure 4) and each combination was repeated 4–8 times. Between different PD/ID combinations, there was always a silent pause of at least 2500 ms. The durations of pulses were 100, 300, 500, 700, 900 and 1200 ms, and the durations of intervals were 500, 1000, 1500, 2000 and 2500 ms. The output intensity at the top of the minishaker was calibrated using a portable digital laser vibrometer PDV-100 (Polytec, Waldbronn, Germany).

### Data evaluation

Data evaluation was carried out using Spike 2 software (Cambridge Electronic Design, Cambridge, UK) and a freeware statistics program (http://faculty.vassar.edu/lowry/VassarStats.html). To show the frequency selectivity, we counted the number of spikes per stimulus for 3–6 pulses of the same frequency for each neuron and presented the data in the form of histograms. To show the temporal selectivity, we constructed the response arrays for each neuron type showing the intensity of the responses either as mean or as peak instantaneous spike rates. We excluded the responses to the 1^st^ repetition of each PD/ID combination and only analysed the responses elicited from the 2^nd^ pulse onward. The reason is that only after the 2^nd^ pulse the neurons started receiving information on both, the PD and the ID. The data are generally presented as means (or as means plus standard errors, since they are the averages of the means of several individual neurons). The number of tested neurons is always given as “N”, whereas the number of data points is given as “n”.

### Neuron terminology

We followed the labelling system of Hedwig [41] and adapted it for the central nervous system of *N. viridula* (for details see: [27]).
